# Wing Phenotypic Variation among *Stomoxys calcitrans* (Diptera: Muscidae) Populations in Thailand

**DOI:** 10.3390/insects13050405

**Published:** 2022-04-23

**Authors:** Tanawat Chaiphongpachara, Gerard Duvallet, Tanasak Changbunjong

**Affiliations:** 1 Department of Public Health and Health Promotion, College of Allied Health Sciences, Suan Sunandha Rajabhat University, Bangkok 10300, Thailand; tanawat.ch@ssru.ac.th; 2 UMR5175, Université Paul-Valéry Montpellier, 34090 Montpellier, France; gerard.duvallet@univ-montp3.fr; 3 Department of Pre-Clinic and Applied Animal Science, Faculty of Veterinary Science, Mahidol University, Nakhon Pathom 73170, Thailand; 4 The Monitoring and Surveillance Center for Zoonotic Diseases in Wildlife and Exotic Animals (MoZWE), Faculty of Veterinary Science, Mahidol University, Nakhon Pathom 73170, Thailand

**Keywords:** geometric morphometrics, phenotype, stable fly, *Stomoxys calcitrans*, Thailand

## Abstract

**Simple Summary:**

The stable fly, *Stomoxys calcitrans* (Diptera: Muscidae), is the predominant *Stomoxys* spp. in Thailand and is considered a pest for livestock, pets, wildlife, and occasionally humans. This study investigated the phenotypic variation in the wing size and shape of *S. calcitrans* populations from different geographical regions in Thailand using landmark-based geometric morphometric analysis. Results showed strong spatial variation in wing shape among *S. calcitrans* populations and thus suggested the existence of phenotypic plasticity in this fly.

**Abstract:**

*Stomoxys calcitrans* (Linnaeus, 1758) (Diptera: Muscidae) is a cosmopolitan hematophagous ectoparasite of veterinary and medical importance. It is an important mechanical vector of several animal pathogens and can cause significant economic losses. However, the morphological variation of this species remains unknown. This study aimed to investigate the phenotypic variation in the wing size and shape of *S. calcitrans* populations in Thailand based on a landmark-based geometric morphometric approach. Specimens were collected from five populations in five geographical regions in Thailand. A total of 490 left wings of *S. calcitrans* (245 female and 245 male individuals) were used for geometric morphometric analysis. Wing size differences were detected between some populations of *S. calcitrans*, whereas wing shape differences were found among populations. Therefore, the phenotypic variation in *S. calcitrans* populations indicated that these populations are adaptive responses to local environmental pressures, suggesting the presence of phenotypic plasticity in this species.

## 1. Introduction

The stable fly, *Stomoxys calcitrans* (Linnaeus, 1758) (Diptera: Muscidae), is one of the 18 species within the subfamily Stomoxyinae and genus *Stomoxys* [1]. This fly is widely distributed worldwide, causing serious health problems to animals, especially livestock, but occasionally humans [2]. Both sexes of *S. calcitrans* are hematophagous and recognized as a mechanical vector of several animal pathogens, including viruses (i.e., equine infectious anemia virus, African swine fever virus, African horse sickness virus, bovine leukemia virus, bovine herpes virus, bluetongue virus, and lumpy skin disease virus [2,3,4,5,6]), protozoa (i.e., *Trypanosoma* spp. and *Besnoitia besnoiti* [7,8]), bacteria (i.e., *Bacillus anthracis* [9] and *Anaplasma marginale* [2]), and helminths (i.e., *Habronema microstoma* [10]). Since *S. calcitrans* can transmit many pathogens, understanding its biology is very important as key knowledge for effective surveillance and control [11].

Phenotypic plasticity, which is also called phenotypic responsiveness, is the capacity of an organism to exhibit distinct phenotypes in response to stimuli from different environments [12]. Environmental heterogeneity affects phenotypic patterns, and these changes can increase the organism’s fitness [13]. Previous studies reported that measurable phenotypic variation could represent the phenotypic plasticity of insect vectors [12,14,15,16]. Currently, wing size and shape are valuable indicators for evaluating the phenotypic variation of insects to adapt to spatially different environments [13,15,17]. Furthermore, variation of the wings of insect vectors is also related to host-feeding sources and flight [12]. In fact, the change in the wing shape of insects can affect their flight capacity, whereas the change in the wing size can be used to estimate the change in body size [18,19]. The longevity of insect vectors has the strongest influence on vectorial capacity due to increased chances of pathogen transmission [20,21]. Barreaux et al. [21] reported the relation between size and longevity in some environments of malaria vector *Anopheles gambiae* (Diptera: Culicidae). Furthermore, Costanzo et al. [22] found the size-fecundity relationship of *Aedes albopictus* (Diptera: Culicidae) when their larvae were reared at high temperatures and low resource levels. Recently, Baleba et al. [23] reported that larval density and substrate quality affected the wing size and shape of *S. calcitrans* and the physical change in wings could significantly affect their flight and dispersion.

Thailand is a Southeast Asian country with a tropical climate [24]. Each geographical region has a varied topography and biodiversity of organisms [24,25]. There are six species of *Stomoxys* spp. distributed in Thailand, but *S. calcitrans* is the most predominant species [26,27]. Muenworn et al. [26] surveyed the distribution of stable flies within six geographical regions in Thailand and indicated that environmental conditions in each collection site were related to the density of flies. However, morphological variation in the wing size and shape of *S. calcitrans* in each geographical region of Thailand remains unknown. The lack of knowledge about insect phenotypic plasticity can be a major obstacle to species identification. Investigators are hesitant about specimens with unusual characteristics, leading to ineffective control measures [14,28].

Geometric morphometrics is a valuable tool and is becoming popular in the study of insect vectors in evaluating size and shape variations. The correlation between size and shape is known as allometry [14]. In insects, wings are the preferred structure for morphometric analyses due to their two-dimensional configurations reducing digitizing error [14]. Geometric morphometric analysis can be performed using three methods: landmark, semi-landmark, and outline-based [14,29,30]. The landmark-based method is most widely used for insect vector species to distinguish morphological closely related species [31,32], identify species [14,32], examine the phenotypic variation among populations [14,33,34], and determine sexual dimorphism [35]. Therefore, this study aimed to estimate the phenotypic variation in the wing size and shape of *S. calcitrans* among five populations in different geographical regions in Thailand based on a landmark-based geometric morphometric approach.

## 2. Materials and Methods

### 2.1. Ethical Statement

All study protocols were conducted according to the guidelines for biomedical research involving animals. This study was approved and endorsed by the Faculty of Veterinary Science, Mahidol University Animal Care and Use Committee (ethical approval no. MU-IACUC 2018/008).

### 2.2. Stable Fly Specimens

**Figure 1:**
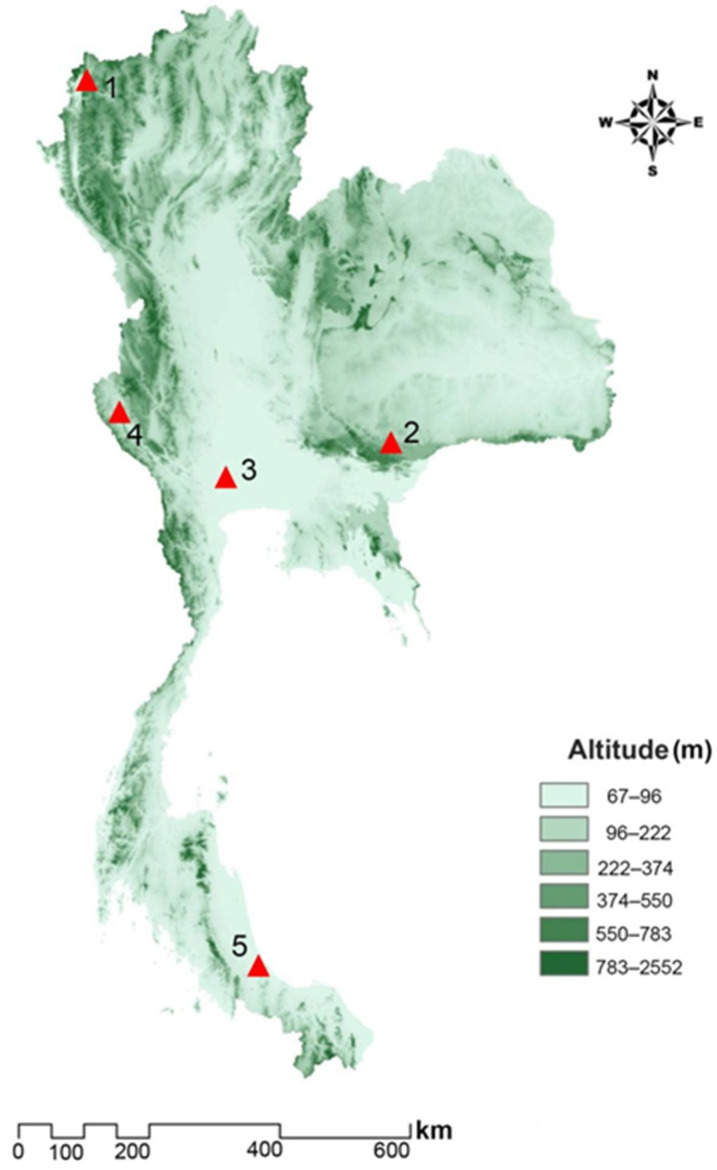
Map of *S.* *calcitrans* populations in Thailand: Mae Hong Son (1), Nakhon Ratchasima (2), Nakhon Pathom (3), Kanchanaburi (4), and Songkhla (5).

Male and female *S. calcitrans* specimens were collected from five populations (also called collection sites) representing five different geographical regions in Thailand, i.e., Mae Hong Son Province (MH; northern region), Nakhon Ratchasima Province (NR; northeastern region), Nakhon Pathom Province (NP; central region), Kanchanaburi Province (KB; western region), and Songkhla Province (SK; southern region; Table 1; Figure 1). Five Nzi traps [36] were used to collect *S. calcitrans* from each population for 2 consecutive days (from 06:00 to 18:00) between February and July 2018. The traps were made locally, using blue and black fabric named Solon^®^ (Bangkok, Thailand) being 100% polyester. The specimens were collected at 2 or 3 h intervals to prevent specimen damage for morphological identification. All specimens were immediately euthanized by freezing at −10 °C, individually placed in 1.5 mL microcentrifuge tubes, and sent to the Vector-Borne Diseases Research Unit, Faculty of Veterinary Science, Mahidol University, Nakhon Pathom, Thailand. The species were identified based on morphological characters by the taxonomic keys of Zumpt [1] and Tumrasvin and Shinonaga [37] under a stereomicroscope (Nikon SMZ745; Nikon Corp., Tokyo, Japan). The specimens were stored at −20 °C until they were used for geometric morphometric analysis.

### 2.3. Sample Preparation and Landmark Digitization

**Figure 2:**
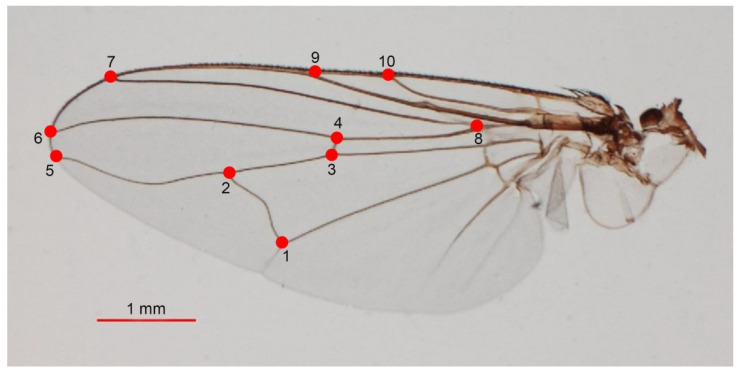
Ten anatomical landmarks selected from the left wing to estimate the phenotypic variation of *S. calcitrans* based on landmark-based geometric morphometric analysis.

The left wings of male and female *S. calcitrans* were detached from the thorax using a sterilized blade and mounted between a microscope slide and cover glass with Hoyer’s medium [31]. Each mounted wing slide was photographed using a digital camera coupled to a stereomicroscope (Nikon AZ 100; Nikon) at 10× magnification and embedded in all wing images at a 1 mm scale unit. Then, ten anatomical landmarks on the intersections of wing veins and intersection with the wing borders [31] were digitized (Figure 2) using XYOM (XY Online Morphometrics) version 2 software [30], which was freely accessed at https://xyom.io/me, accessed on 15 February 2022. The geometric and statistical analyses and graphic outputs were also performed by XYOM.

### 2.4. Repeatability and Allometry

Before wing size and shape analyses, repeatability and allometry were examined. A repeatability test is important to assess the accuracy of landmark digitization based on comparing two sets of wing images. Ten images per population of male and female *S. calcitrans* were randomly selected and digitized twice by the same user. The repeatability index was computed based on the Procrustes analysis of variance (ANOVA) method to examine the measurement error of landmark digitization [38]. As for allometry, the estimation of the allometric effect is also important to assess the effect of the wing size on wing shape variation. Linear regression based on the first (shape-derived) discriminant factor (DF) on wing size was used in this investigation and estimated by the determination coefficient (r^2^).

### 2.5. Wing Size Analyses

Centroid size (CS) was calculated from the square root of the sum of squared distances between the centroid and each landmark to represent the global wing size of *S. calcitrans* to determine size variation among different populations [39]. Graphic quantile boxes were built to display wing CS variations of *S. calcitrans* in each population. Differences in the average wing CS of male and female *S. calcitrans* between populations were compared using one-way ANOVA followed by Bonferroni post hoc test. A nonparametric procedure (1000 permutations) was used to estimate statistical significance at *p* < 0.05.

### 2.6. Wing Shape Analyses

The wing shape variables were obtained through a Procrustes superimposition according to the Generalized Procrustes Analysis. The principal components of shape variables were used as final shape variables for wing shape analysis. Wing shape variations of male and female *S. calcitrans* among populations were estimated by discriminant analysis according to the first two DFs and illustrated by factor maps. The Mahalanobis distance was calculated to estimate the metric distance of shape divergence between groups. The statistical significance of average wing shape differences based on Mahalanobis distances of male and female *S. calcitrans* between populations was calculated by a nonparametric permutation test (1000 permutations) at *p* < 0.05. Furthermore, to assess the relationships of wing shape of *S. calcitrans* among populations, a UPGMA algorithm based on the Mahalanobis distances was used to illustrate a hierarchical clustering tree. Branch support was estimated based on 1000 bootstrap replicates for each data set.

### 2.7. Validated Classification

A cross-validated classification test was used to analyze the accuracy of wing size and shape that may be specific to different populations. Each individual sample was sequentially removed from the total sample and assigned to the most likely (for size) and closest group (for shape) based on the maximum likelihood method and Mahalanobis distance, respectively.

## 3. Results

In this study, 490 *S. calcitrans* (245 female and 245 male individuals) collected from five populations representing five geographical regions in Thailand were used to examine the phenotypic variation of wing size and shape using the landmark-based geometric morphometric method.

### 3.1. Repeatability of Wing Image

The quality in digitizing landmarks of our wing image set based on testing repeatability revealed that the repeatability score of male and female *S. calcitrans* was high (95% for shape). Meanwhile, the measurement error was low (5% for shape).

### 3.2. Allometric Effect

**Figure 3:**
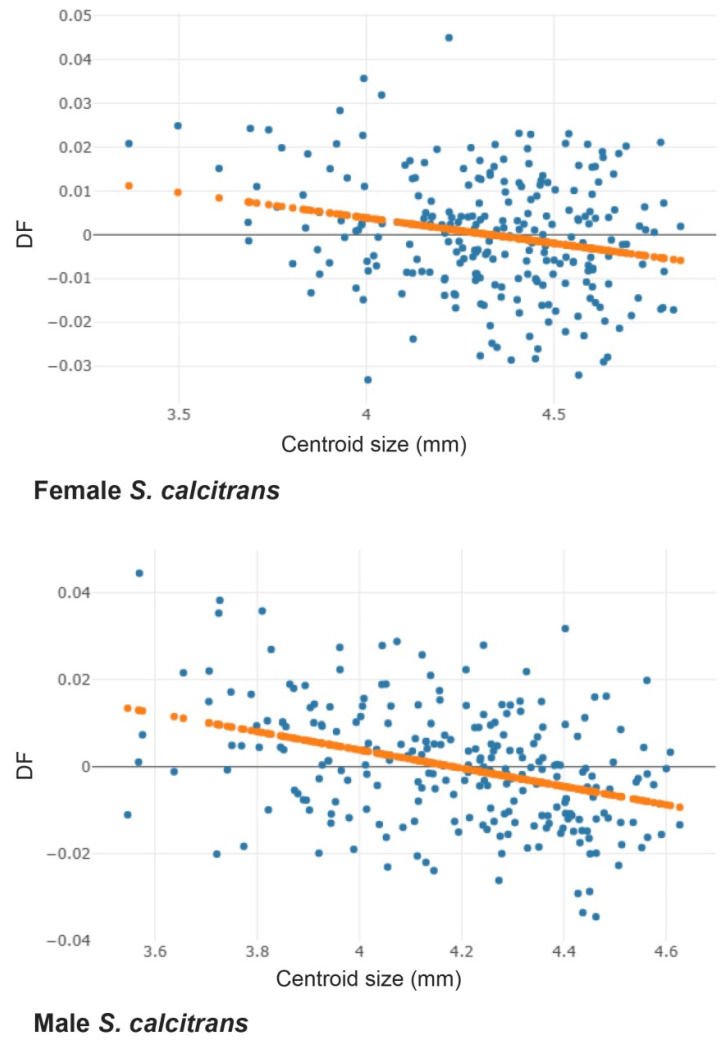
Linear regression between wing size (CS) and shape (DF) of female (**top**) and male (**bottom**) *S.* *calcitrans*. Orange dotted lines indicate linear regression prediction.

An analysis of the relationship between the size and shape of male and female *S. calcitrans* showed that wing size variation was significantly correlated to wing shape changes (*p* < 0.05; Figure 3). Linear regression prediction revealed a negative correlation, meaning a smaller wing size correlated with a greater difference in wing shape (r^2^ = 15.0% for females and r^2^ = 24.0% for males).

### 3.3. Wing Size Variation

**Figure 4:**
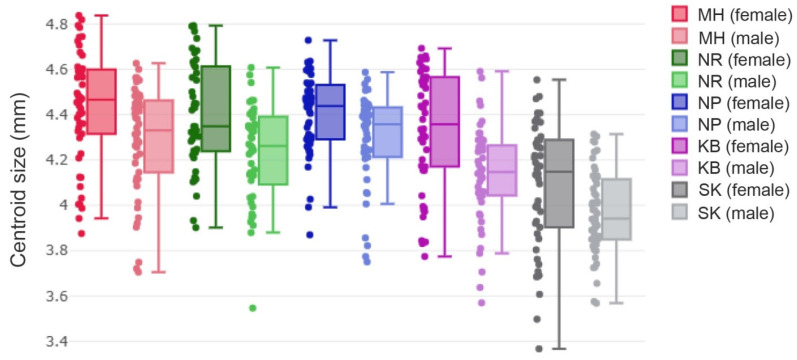
Quantile boxes of wing CS variations of male and female *S.* *calcitrans* populations. The horizontal line crossing each box is the median separating the 25th and 75th quartiles.

Overall, the wing CS of female *S. calcitrans* (ranged from 4.09 to 4.43 mm) was larger than that of males (ranged from 3.97 to 4.30 mm; Figure 4). The analysis of the wing CS variation of female *S. calcitrans* among different populations in Thailand showed that the MH population was the largest (4.43 mm), followed by NR and NP (4.40 mm), KB (4.34 mm), and SK (4.09 mm), whereas the wing CS variation of male *S. calcitrans* showed that the NP population was the largest (4.30 mm), followed by MH (4.29 mm), NR (4.23 mm), KB (4.14 mm), and SK (3.97 mm; Table 2). A statistically significant difference in the wing CS of female *S. calcitrans* was found between the SK population and all population groups (*p* < 0.05), whereas statistical significance in the wing CS of male *S. calcitrans* was found between the SK population and all population groups (*p* < 0.05), MH and KB (*p* < 0.05), and NP and KB (*p* < 0.05).

### 3.4. Wing Shape Variation

**Figure 5:**
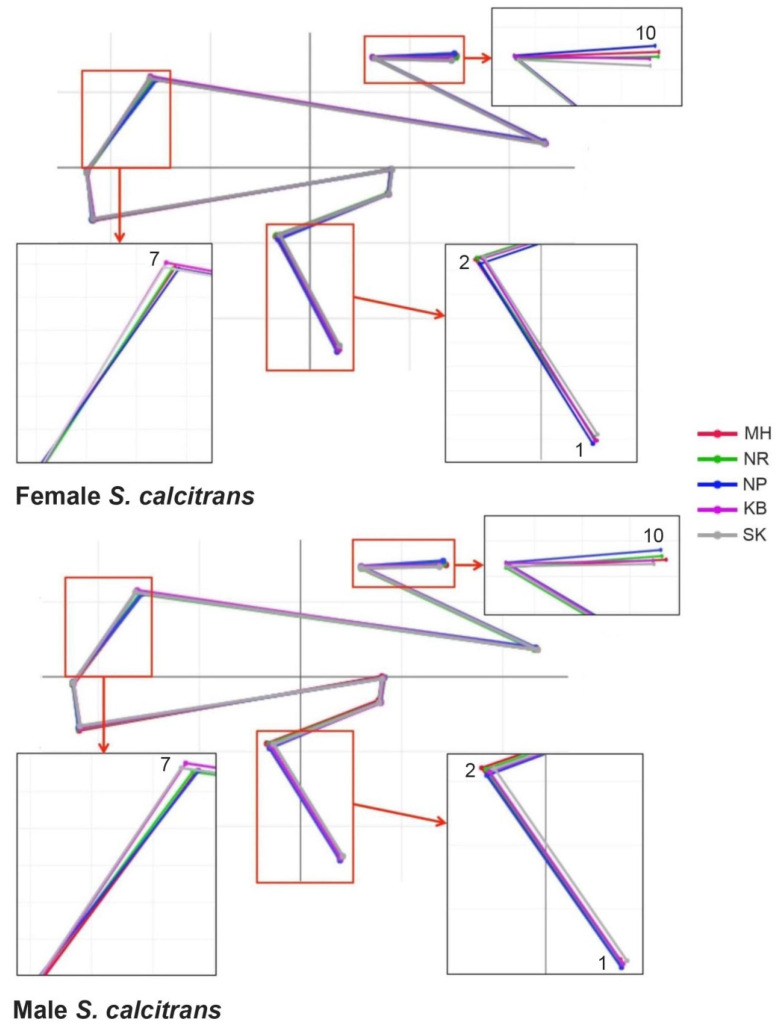
Superposition of the aligned mean anatomical landmark positions of female (**top**) and male (**bottom**) *S.* *calcitrans* populations. Enlarged images in small frames showed the parts of wing construction where variation occurred.

After the generalized Procrustes analysis, graphic constructions of the wing shape of female and male *S. calcitrans* were built from the superimposition of aligned mean configurations. These graphic wing constructions revealed the most visible displacement at 1, 2, 7, and 10 landmark positions (Figure 5).

**Figure 6:**
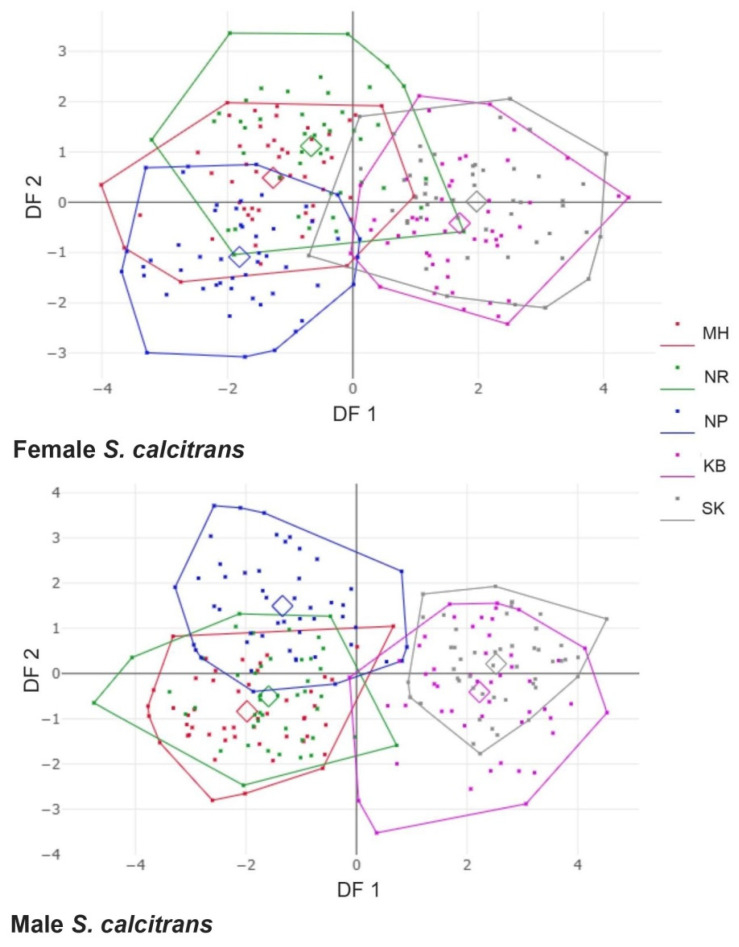
Factor maps based on discriminant analysis in wing shape variation of female (**top**) and male (**bottom**) *S.* *calcitrans* populations. Each point in a polygon represents an individual wing sample, and small squares in a polygon represent the position of the mean group. The horizontal axis was the first DF (DF1), whereas the vertical axis was the second DF (DF2).

**Figure 7:**
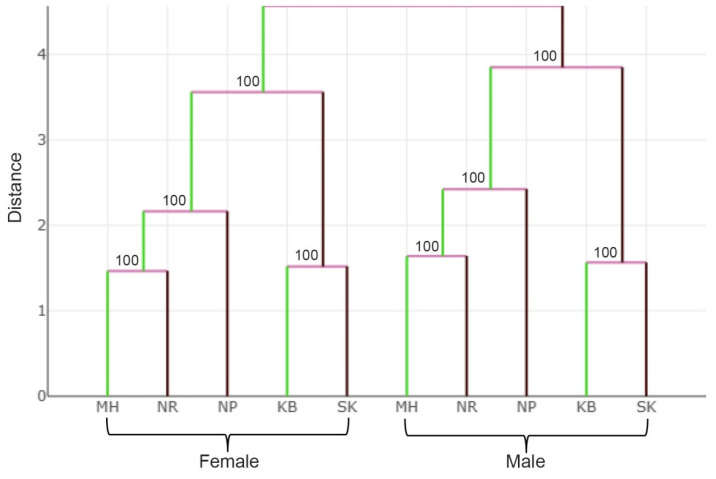
Hierarchical clustering tree based on Mahalanobis distances of female and male *S.* *calcitrans* populations. Numbers at the nodes indicate the percentages of bootstrap values based on 1000 bootstraps.

The analysis of the wing shape variations among *S. calcitrans* populations in factor maps based on discriminant analysis defined by DF axes showed that the first two DF axes accounted for 89% of the total wing shape variation for female *S. calcitrans* (DF1 = 73% and DF2 = 16%) and 91% for male *S. calcitrans* (DF1 = 77% and DF2 = 14%; Figure 6). All female *S. calcitrans* populations represented overlapping, and no distinct populations were separated from each other. In contrast, all-male populations represented a majority overlap, and nearly all populations were not separated, except for the SK population separated from MH, NR, and NP (Figure 6). However, comparing pairwise Mahalanobis distances of male and female *S. calcitrans* between populations showed statistically significant differences (*p* < 0.05, Table 3). A hierarchical clustering tree based on Mahalanobis distances of *S. calcitrans* populations showed the same pattern based on the wing shape between females and males (Figure 7). The wing shape of MH was similar to NR than NP, whereas KB was similar to SK and separated from other populations supported by 100% bootstrap values. The tree also showed clear wing shape differences between females and males based on group separation.

Cross-validated classification yielded low and high correctly assigned scores for wing size (0–58%) and shape (51.11–80%), respectively (Table 4).

## 4. Discussion

The study of phenotypic variation is important to understand the influence of environmental and/or genetic factors in a population. This study investigated wing size and shape variations among *S. calcitrans* populations representing five different geographical regions in Thailand based on a geometric morphometric approach. The wing size of female and male *S. calcitrans* in SK was significantly smaller than in other populations. SK is a coastal province in Thailand located near the Gulf of Thailand. The influence of coastal climates may affect the wing size of *S. calcitrans*. Previous studies indicated that some insects in coastal areas have smaller wing sizes than those in mainland areas. Sumruayphol et al. [40] studied *Phlebotomus stantoni* (Diptera: Psychodidae) from different provinces in Thailand and found that the wing size of the population in Lang Ga Jiew Island, Chumphon Province, was the smallest. Demari-Silva et al. [41] found that *Culex coronator* (Diptera: Culicidae) populations in Brazil’s Rio de Janeiro Municipality lowland coastal areas had significantly smaller wing sizes than in other areas. However, some dipteran insects exhibited the opposite effect depending on their suitability for life in coastal areas. Chaiphongpachara et al. [17] found that the average wing size of female *Aedes aegypti* (Diptera: Culicidae) in coastal areas was significantly larger than in the residential and cultivated areas in Samut Songkhram Province, Thailand.

Furthermore, high-quality food sources and their population density in each area are key factors in the wing size changes of insects [42]. High food quality and suitable population density result in larger wing sizes of insects than in food-poor areas and high population density [23,42]. Consequently, different wing sizes in each area can account for the degree of suitability of their habitat [23]. In this study, the habitat of *S. calcitrans* in southern Thailand was a zoo located in an urban area. Changbunjong et al. [27] studied stomoxyine flies across Thailand and found that zoos have a denser *S. calcitrans* population than livestock farms. Although zoos have a wide variety of animal hosts, they have limited spatial restrictions and are located in urban areas, resulting in a low level of biodiversity of stomoxyine flies and making the *S. calcitrans* population the most predominant species in zoos without competing species [27]. 

Wing shape is related to flying capability [43]. This capacity of insect vectors is used for host-seeking. Data analyses revealed that the wing shape of female and male *S. calcitrans* indicates the variation between different populations of Thailand. Wing shape differences are caused by different environmental influences. Previous studies reported that certain environmental factors affect the insect wing shape [15,44,45]. Phanitchat et al. [46] studied the change in *Ae. albopictus* wings with temperature and found that wing shape changed with increasing temperature. In addition, larval density and the nature of the developmental substrate are also natural factors to influence the wing shape of *S. calcitrans* [23]. Meanwhile, the seasonal variations could have an impact on wing shape as well as wing size of *S. calcitrans* in our study. Prudhomme et al. [34] found that seasonal environments affect wing shape and size variations in *Phlebotomus ariasi* (Diptera: Psychodidae). However, environmental factors can influence wing size much more than wing shape [14].

A hierarchical clustering tree revealed the proximity of wing shape among *S. calcitrans* populations in Thailand. It was supposed that wing shape variation might be related to the altitude of the areas. The altitude causes different ecosystems due to different environmental factors, such as levels of sunlight, temperature, wind, relative humidity, host species, and resident plant species [47]. The wing shapes of northern (MH) and northeastern (NR) populations were similar. These results may be because both sites are located at high altitudes (452 and 498 m). The wing shapes of western (KB) and southern (SK) populations were similar. Both sites are moderate-altitude areas (174 and 112 m). The wing shape of the central (NP) population as a low-altitude area (11 m) was sandwiched between those populations. These results were consistent with Lorenz et al. [47] that wing shapes of the malaria vector *Anopheles cruzii* (Diptera: Culicidae) in Brazil were distinct between lowland (altitude of 5–20 m) and hilltop (altitude of 81–263 m) populations. The comparison of pairwise Mahalanobis distances in this study indicated that populations in southern regions had a different wing shape than other regions. The southern region of Thailand is the most different area from other regions. This region is along the Andaman Sea and the Gulf of Thailand and has only two seasons, summer and rainy, whereas other regions have three seasons, summer, rainy, and winter [48]. This phenomenon might explain the highest wing shape variation among populations of *S. calcitrans* in Thailand. The results from the hierarchical clustering tree also revealed a sexual dimorphism in the wing shape of *S. calcitrans* in Thailand. These results indicated that the phenotypic expression of wing shape was a sex-specific difference. These results were consistent with a previous report on other *Stomoxys* spp. (*Stomoxys indicus* (Diptera: Muscidae), *Stomoxys* *pullus* (Diptera: Muscidae), and *Stomoxys* *uruma* (Diptera: Muscidae)) [31]. The sexual dimorphism of wing shape was also reported in other insect vectors, such as mosquitoes of the genera *Aedes* (*Ae. aegypti* and *Ae. albopictus*), *Anopheles* (*Anopheles* *albitarsis* (Diptera: Culicidae), *Anopheles* *cruzii* (Diptera: Culicidae), *Anopheles* *homunculus* (Diptera: Culicidae), *Anopheles* *strode* (Diptera: Culicidae), and *Anopheles* *triannulatus* (Diptera: Culicidae)), *Culex* (*Culex* *quinquefasciatus* (Diptera: Culicidae) and *Culex* *nigripalpus* (Diptera: Culicidae)), and *Ochlerotatus* (*Ochlerotatus* *scapularis* (Diptera: Culicidae)) [49] and the biting fly *Haematobosca aberrans* (Diptera: Muscidae) [35]. Furthermore, sexual shape dimorphism in this study was correlated with sexual size dimorphism (female, 15%; male, 24%). This suggested that allometry is an important factor in sexual shape dimorphism in *S. calcitrans* wings.

In this study, cross-validated classification scores showed that the wing shape of *S. calcitrans* was more specific to each population than wing size (18.37% for size and 61.63% for shape). These results indicated that wing shapes of *S. calcitrans* expressed phenotypes in response to the unique topography of each geographical region more than wing size. Wing size is a very sensitive factor to the environment compared to wing shape [50]. Therefore, wing size was highly variable and did not reflect interregional specificity in this study. All results were supported by linear regression prediction of the relationship between size and shape based on allometry. Linear prediction explained that a smaller wing size correlated with a greater difference in wing shape. This relationship can explain the natural pressures from environmental differences. However, non-allometric effects, such as flight behavior and mating systems, may be related to variations in wing shape [51].

Although phenotypic variation results from environmental factors, it can also result from genetic factors [14]. However, a previous study on genetic differences based on cytochrome *c* oxidase subunit I in *S. calcitrans* populations from Thailand showed that low intraspecific divergence ranged from 0 to 3.2% (mean = 0.8%) [52]. In addition, a study of the population structure of *S. calcitrans* from nine provinces of Thailand using allele variation frequencies of isozymes demonstrated no significant genetic difference among the nine populations [53]. Hence, these results indicated that the phenotypic plasticity of the *S. calcitrans* population in this study might not be related to genetic variation. In contrast, genetic factors affected the phenotypic variation of some insect species, such as *Drosophila melanogaster* (Diptera: Drosophilidae) [54].

## 5. Conclusions

This study provided phenotypic information on *S. calcitrans* populations in Thailand based on a landmark-based geometric morphometric analysis of wing size and shape variations. Results showed that wing size differences were detected between some populations of *S. calcitrans*, whereas wing shape differences were detected among populations. Wing size variation had a significant effect on wing shape variation. These results indicated that phenotypic variation in *S. calcitrans* is an adaptive response to local environmental pressures in the study areas. It was speculated that the effects of this physical change might affect the biology of this species in the aspects of dispersion and spread of the diseases. However, the relationship between phenotypic variation and environmental factors and/or pathogen transmission should be considered in further studies.

## Figures and Tables

**Table 1:** Population, date, and number (n) of wing images of *S.* *calcitrans* used for the landmark-based geometric morphometric analysis.

Population	Code	Region	Date	Biotope	Altitude	Coordinates (Lat/Long)	n
Mae Hong Son	MH	Northern	July	Beef cattle farm located in rural area	452	19°31′45″ N, 98°04′48″ E	Female 50, Male 50
Nakhon Ratchasima	NR	Northeastern	February	Beef cattle and buffalo farm located in rural area	498	14°22′23″ N, 101°44′51″ E	Female 45, Male 45
Nakhon Pathom	NP	Central	May	Beef cattle farm located in urban area	11	14°01′10″ N, 99°57′37″ E	Female 50, Male 50
Kanchanaburi	KB	Western	March	Beef cattle farm located in rural area	174	14°39′28″ N, 98°32′19″ E	Female 50, Male 50
Songkhla	SK	Southern	February	Zoo located in urban area	112	07°08′26″ N, 100°36′20″ E	Female 50, Male 50

**Table 2:** Mean CS of male and female *S.* *calcitrans* populations and statistically significant differences.

Population	n	Mean (mm)	(Min–Max)	Variance	SD	SE
Female						
MH	50	4.43 ^a^	3.88−4.84	0.06	0.25	0.04
NR	45	4.40 ^ac^	3.90−4.79	0.05	0.24	0.04
NP	50	4.40 ^ad^	3.87−4.73	0.03	0.18	0.02
KB	50	4.34 ^ab^	3.77−4.69	0.07	0.26	0.04
SK	50	4.09 ^h^	3.37−4.55	0.08	0.28	0.04
Male						
MH	50	4.29 ^be^	3.71−4.63	0.05	0.23	0.03
NR	45	4.23 ^efg^	3.55−4.61	0.05	0.22	0.03
NP	50	4.30 ^bcdf^	3.75−4.59	0.04	0.20	0.03
KB	50	4.14 ^gh^	3.57−4.59	0.05	0.21	0.03
SK	50	3.97 ^i^	3.57−4.31	0.03	0.18	0.03

Statistically significant differences (*p* < 0.05) are indicated by different letters.

**Table 3:** Mahalanobis distances (below diagonal) and *p*-values (above diagonal) among the wing shapes of female and male *S.* *calcitrans* populations.

Population	MH	NR	NP	KB	SK
Female					
MH	-	0.005	<0.001	<0.001	<0.001
NR	1.55	-	<0.001	<0.001	<0.001
NP	1.94	2.51	-	<0.001	<0.001
KB	3.22	2.96	3.69	-	<0.001
SK	3.45	2.99	3.98	1.41	-
Male					
MH	-	<0.001	<0.001	<0.001	<0.001
NR	1.68	-	<0.001	<0.001	<0.001
NP	2.52	2.26	-	<0.001	<0.001
KB	4.38	3.99	4.08	-	<0.001
SK	4.69	4.35	4.17	1.61	-

**Table 4:** Percentage of correctly assigned individuals based on the cross-validated classification of the wing size and shape of female and male *S. calcitrans* populations.

Population	Size		Shape	
	% Correctly Assigned Individuals	No. of Correctly Assigned Individuals/Total Numbers	% Correctly Assigned Individuals	No. of Correctly Assigned Individuals/Total Numbers
Female				
MH	58	29/50	52	26/50
NR	0	0/45	51.11	23/45
NP	2	1/50	70	35/50
KB	6	3/50	66	33/50
SK	16	8/50	54	27/50
Male				
MH	12	6/50	58	29/50
NR	13.33	6/45	53.33	24/45
NP	4	2/50	72	36/50
KB	18	9/50	58	29/50
SK	52	26/50	80	40/50
Total	18.37	90/490	61.63	302/490

## Data Availability

The data presented in this study are available on request from the corresponding author.
